# Evaluation of Antibiotic Resistance Pattern in Dental Bacteremia Detected by Multiplex PCR Technique

**DOI:** 10.1155/2020/9502959

**Published:** 2020-10-01

**Authors:** Fahimeh Rezazadeh, Azita Azad, Ali Khorami, Farzan Modaresi, Zahra Rezaie

**Affiliations:** ^1^Oral and Dental Disease Research Center, Department of Oral and Maxillofacial Medicine, School of Dentistry, Shiraz University of Medical Sciences, Shiraz, Iran; ^2^Student Research Committee, School of Dentistry, Shiraz University of Medical Sciences, Shiraz, Iran; ^3^Departments of Microbiology, Advanced Medical Sciences and Technology, and Central Laboratory Research, Jahrom University of Medical Sciences, Jahrom, Iran; ^4^Student Research Committee, Jahrom University of Medical Sciences, Jahrom, Iran

## Abstract

The aim of this study was to detect oral bacteremia and offer the antibiotic resistance patterns. Bacterial resistance pattern was evaluated in 50 patients. A spectrophotometer device equipped with UV and electrophoresis of the extracted samples on agarose gel for antibiogram test were used. PCR test 15 minutes after tooth extraction showed that bacterial strains were extracted from 16 patients. *Lactobacillus*, *Enterococcus faecalis (E. faecalis)*, *Streptococcus sanguinis (S. sanguinis)*, *Streptococcus salivarius (S. salivarius)*, and *Streptococcus mutans* (*S. mutans)* were extracted from 5, 4, 4, 4, and 6 patients. 100% of *Lactobacillus*, *E. faecalis*, *S. sanguinis*, *S. salivarius*, and *S. mutans* were sensitive to tigecycline. Most of the *Lactobacillus* antibiotic resistance was against tetracycline and ciprofloxacin. Antibiotic resistance in *S. salivarius* was observed in 75% of the cases against piperacillin-tazobactam, ciprofloxacin, and cefotaxime, while in *S. mutans* was 84% of the cases against ceftriaxone. The results of the current study showed that tooth extraction causes bacteremia before, during, and after tooth extraction. Generally, the highest antibiotic resistance occurred against tetracycline, ciprofloxacin, and ampicillin-sulbactam. In most cases, the bacteria showed partial resistance to these antibiotics; however, tigecycline showed 100% efficacy on all types of bacteria. *Streptococcus* strains (*salivarius*, *mutans*, and *sanguinis*) were sensitive to most of the antibiotics while antibiotic sensitivity was less evident in *Lactobacillus* and *E. faecalis*. Antibiotic resistance has become a critical issue, since it leads to treatment failure when there is a need for antibiotic therapy.

## 1. Introduction

Transient bacteremia (TB), the presence of bacteria in blood circulation, is anticipated to happen as a result of dental and periodontal manipulation. According to recent studies, the prevalence of transient bacteria after tooth extraction varies between 30 and 60% in adults and 33 and 80% in children [1–3]. Infectious endocarditis, organ transplant failures and various infections are some side effects of transient bacteremia in immunosuppressed patients [4, 5]. In order for this situation to be controlled and prevented, antibacterial mouth rinses and systemic antibiotics can be prescribed [4, 6]. Prophylactic antibiotics are given to patients with cardiovascular diseases, patients who undergo hemodialysis, or immunocompromised patients such as those who have received the organ transplants, in order to reduce the risk of serious infections as a consequence of bacteremia [7]. The fundamental approach in treating infection is to choose a high-efficacy antibiotic [6]. Unfortunately, inappropriate and over-use of antibiotics have led to the elimination of sensitive microorganisms, which offers an environment for resistant bacteria to survive [8], leading to reduced efficacy of some antibiotics. Antibiotic resistance has become an important issue, since it causes treatment failures when it is necessary [9]. According to researches, antibiotic resistance is most prevalent in developing countries. Therefore, annual evaluations of drug resistance pattern are essential, regarding the microbial agents and various drug resistance patterns in different regions [10]. Bacteria are capable to grow and proliferate in an environment that has essential nutrition, water, mineral, and organic agents. Such environment is referred to as a culture medium where bacteria are cultured [11]. The gold standard for diagnosis of bacteremia is culturing technique [12]. Blood is the best culture media for bacterial proliferation. Many culture-based microbiological techniques such as quantitative, semi-quantitative (lyse-centrifuge and lyse-filtration), and CO production by cultured bacteria are available for examination of blood after tooth extraction [13]. Molecular techniques like polymerase chain reaction (PCR) with higher sensitivity and specificity can be helpful in the detection of bacteremia. The spontaneous evaluation of multiple specimens is possible via Multiplex PCR, with higher sensitivity and specificity. With properties of new PCR methods in better detection of bacteria and pathogenic strains, and appropriate prescription of specific antibiotics, drug resistance in pathogenic bacteria might potentially decrease [14, 15]. The use of the Multiplex PCR method is mentioned in some researchers, but to the best of our knowledge, there is no literature available on the detection of oral bacteria via the Multiplex PCR method.

## 2. Materials and Methods

In this cross-sectional descriptive study, bacterial resistance pattern was evaluated in 50 patients, ages from 18 to 45 years old, including 32 males and 18 females, between April 2014 and October 2018 with the need for tooth extraction under general anesthesia who visited the health centers of Jahrom University of Medical Sciences. All patients were single dental extraction without any dental or oral pathology interferences.

**Figure 1:**
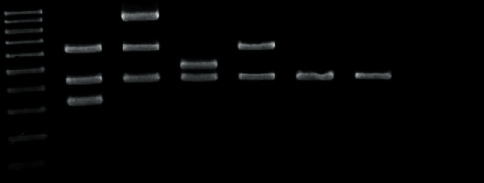
Multiplex PCR of all five bacteria using ladder 100 bp. Triplicate experimental performed.

**Figure 2:**
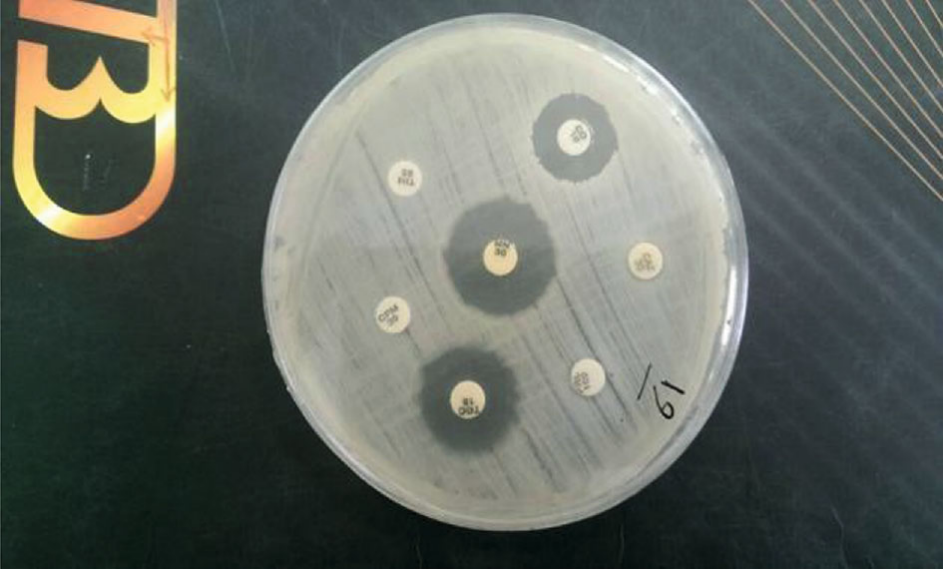
Disk diffusion test for antibiotics.

The exclusion criteria for this study were unwillingness to participate in the study, history of antibiotic intake (including prophylactic regimens prior to surgical procedures), immunodeficiency (acquired or congenital), and manifestations of systemic diseases. This study was approved by the local ethics committee of Shiraz University of Medical Sciences, and an informed consent was obtained from each individual who participated in this study. In order to detect the prevalence of transient bacteremia after tooth extraction, a 10 ml sample of venous blood was taken from each patient, in a citrate-containing test tube (after nasotracheal intubation and before local anesthesia injection with adrenaline and articaine). The same volume of blood sample was also taken 30 seconds and 15 minutes after final tooth extraction. Sample transformation to the microbiology laboratory of Jahrom University of Medical Sciences and the preparation of the cultures were performed based on routine laboratory protocols. In order for bacteria to grow and to evaluate colony properties, blood samples from each time and interval were cultured on blood agar, mitis salivarius agar, and bile esculin agar. After the colonies were observed in the plate, microscopic slides were prepared and gram staining was performed for each slide. Conventional microbiological and chemical tests were performed to identify microorganisms. Sticky, dark, smooth *Streptococcus mutans (S. mutans)* colonies were detected in the mitis salivarius media after 48 hours. *Enterococcus faecalis (E. faecalis)* colonies in bile esculin agar medium caused brown discoloration in the 2 culture media. An alternative method of culturing was also performed for the extraction to be highly accurate. For *Streptococcus* detection and extraction, biochemical oxidase tests, 4% medium culture and 5.6% NaCl, acetoin production (VP), esculin hydrolysis and blood agar hemolysis, and a phenol red-based medium with sugar were used for carbohydrate metabolism. For *Lactobacillus* detection and evaluation of carbohydrate metabolism, sugar was added to a meat and glucose-free agar and a bacterial culture was performed following the addition of phenol red. In the SIM medium, yolk and gelatin were used for lecithinase, movement, and gelatinase tests. Precautions taken to avoid the risk and contamination of samples in the processing of PCR in the laboratory were taken by separating pre- and postamplification areas, which is key to preventing contamination. The procedure begins by preparing the PCR master mix in a template-free room, using reagents that never come into contact with potential sources of contamination. Maintain a separate area for analyzing PCR amplicons. A Fermentas deoxyribonucleic acid (DNA) extraction kit (Lithuania) was used in this research. It consists of 3 vials including the lysis solutions for cell lyses, precipitation solution for DNA precipitation, and NaCl solution for ion adjustment. For quantitative and qualitative examinations of the extracted genome DNA (amount of DNA), a spectrophotometer equipped with UV (260 and 280 nm wavelengths) and electrophoresis of the extracted samples on agarose gel was used. Relative primer sequences of authentic researches were extracted from the available data on the databases such as Genbanka (Table 1). Samples were put in the GC1-96 thermos cycle device to undergo PCR procedures. The DNA was detected following the electrophoresis on agarose gel or poly-acrylic amid and ethidium bromide (Figure 1). Agar disk diffusion was done on a molar Hinton agar for antibiogram test (Figure 2). Some bacterial colonies were picked by straight wire (no loop) instrument after bacterial isolation and solved in physiologic serum. The solution was transferred into the molar Hinton agar. The plate was fully meadow kill cultured by a swab. Antibiogram discs were put on the culture medium; the plate was closed, and incubated at 37°C. The plates were examined under a lamp after 24 hours. The diameter of the inhibition zone was measured by a ruler, and the results of antibiogram test were reported as susceptible, resistant, or intermediate, based on the CLSI 2018 Table. 20 antibiotics were chosen based on the CLSI 2018 protocol (Table 2). The descriptive statistics were reported.

## 3. Results

In total, 32 men (64%) and 18 women (36%) with an average age of 15.25 years (24.09 ± 8.94 and 26.22 ± 8.03, respectively) participated in this study.

### 3.1. Culture Test before Tooth Extraction

No bacteria were detected in the cultures of preextraction samples.

### 3.2. Culture Test 30 Seconds after Tooth Extraction

31 patients (62%) exhibited positive bacterial culture during tooth extraction. According to blood culture, 7 patients had more than one strain of bacteria and 24 patients had only one strain.

### 3.3. Culture Test 15 Minutes after Tooth Extraction

Ten patients (20%) had positive culture 15 seconds after tooth extraction, and 4 *Lactobacillus*, 3 *E. faecalis*, 2 S*. sanguinis*, and 2 *S. mutans* cultures were observed. Only one patient had *Lactobacillus* and *S. mutans* in the blood sample, simultaneously.

### 3.4. PCR Test before Tooth Extraction

PCR showed that 5 cases (10%) had positive bacteremia: 3 *E. faecalis*, 1 S*. sanguinis*, 1 *S. salivarius*, and 1 *S. mutans*.

### 3.5. PCR Test 30 Seconds after Tooth Extraction

39 patients (78%) had positive bacteremia 30 seconds after tooth extraction. *Lactobacillus* was extracted from 20 samples; *E. faecalis*, *S. sanguinis*, *S. salivarius*, and *S. mutans* were extracted from 14, 11, 15, and 22 samples, respectively.

### 3.6. PCR Test 15 Minutes after Tooth Extraction

Bacterial strains were extracted from 16 (32%) patients. *Lactobacillus*, *E. faecalis*, *S. sanguinis*, *S. salivarius*, and *S. mutans* were extracted from 5, 4, 4, 4, and 6 samples, respectively.

### 3.7. Antibiotic Resistance Pattern 30 Seconds after Tooth Extraction

20 cases (40%) were detected to have *Lactobacillus* in their PCR, and the highest sensitivity was related to imipenem and tigecycline (100% of the bacteria were sensitive to these antibiotics). The most *Lactobacillus* antibiotic resistance was against tetracycline. Ten out of 20 samples (50%) were resistant to tetracycline.

15 cases (30%) were detected to have *E. faecalis* in their PCR, and the highest sensitivity was related to piperacillin-tazobactam and tigecycline 100% of the bacteria was sensitive to these antibiotics. The most *E. faecalis* antibiotic resistance was against tetracycline, ampicillin sulbactam, and ciprofloxacin. Five out of 15 samples (33%) were resistant to these antibiotics.

11 cases were detected to have *S. sanguinis*. It was sensitive to most of the antibiotics (most of the antibiotics showed more than 90% efficacy on these bacteria). Cefepime, piperacillin-tazobactam, and tigecycline showed 100% efficacy in eliminating *S. sanguinis*. Antibiotic resistance in this strain was observed in few cases, and the highest resistance was to amikacin which was observed in 2 out of 11 cases (18%).

15 cases (30%) were detected to have *S. salivarius* in their PCR, and the highest sensitivity was related to tigecycline (100%); imipenem and ceftazidime showed a 90% efficacy on *S. salivarius*. Antibiotic resistance in this strain was observed in few cases, and the highest resistance was to rifampin, ampicillin, tazobactam, and tetracycline. Five out of 15 cases (33%) were resistant to this antibiotic.

22 cases (44%) were detected to have *S. mutans* in their PCR, and the highest sensitivity was related to tigecycline (100%); imipenem and ertapenem showed a 90% efficacy on *S. salivarius*. The highest resistance was to colistin (10 cases, 45%) and tetracycline (12 cases, 52%).

### 3.8. Antibiotic Resistance Pattern 15 Minutes after Tooth Extraction

Five cases (10%) were detected to have *Lactobacillus* in their PCR, and highest sensitivity was related to tigecycline (100% of the bacteria were sensitive to these antibiotics). The most *Lactobacillus* antibiotic resistance was against tetracycline and ciprofloxacin. Three out of 5 cases (60%) were resistant to these antibiotics.

Four cases (8%) were detected to have *E. faecalis* in their PCR, and highest sensitivity was related to piperacillin-tazobactam and tigecycline. 100% of the bacteria were sensitive to these antibiotics. Three out of 4 cases (75%) were resistant to these antibiotics.

Three cases (8%) were detected to have *S. sanguinis*. It was sensitive to most of the antibiotics (most of the antibiotics showed more than 90% efficacy on these bacteria). Cefepime, piperacillin-tazobactam, and tigecycline showed 100% efficacy in elimination of *S. sanguinis*. Antibiotic resistance in this strain was observed in only few cases.

Four cases (8%) were detected to have *S. salivarius* in their PCR, and highest sensitivity was related to tigecycline (100%). Antibiotic resistance in this strain was observed in 75% of the cases against piperacillin-tazobactam, ciprofloxacin, and cefotaxime.

Six cases (4412 were detected to have *S. mutans* in their PCR, and highest sensitivity was related to tigecycline (100%). Antibiotic resistance in this strain was observed in 84% of the cases against ceftriaxone.

## 4. Discussion

Human oral cavity is colonized by a different bacterial flora from other anatomic areas of the body. More than 200 different organisms are detected in the oral cavity [1]. Bacterial colonization is specifically related to gingival sulcus around the tooth; wherein in total health, a thin, nonkeratinized epithelial mucosa separated potential pathogenic microorganisms from basal membrane, lymphatic, and general blood circulation [2]. It was calculated that in healthy adults, superficial gingival tissues (pockets) consist of 4 cm^2^ of the gingival subicular epithelium. In times of gingival or periodontal infections, these superficial areas can dramatically increase in size and a compressed mass of microorganisms can easily enter this infectious high vascular tissue [3]. In addition, recent studies have shown than some of these organisms can invade the space between epithelial tissues and deep periodontal fibers [4]. While bacteremia normally depends on tracheal intubation [5], other factors such as oral health, number of extracted teeth, and type of anesthesia (general anesthesia) can affect the prevalence of bacteremia [6]. Generally, it is believed that bacteremia is related to invasion, duration of tooth surgery, and tooth extraction [7]. Tooth extraction is a common dental procedure, causing bacteremia in 10-92% of the people. It should be kept in mind that routine dental care might harm the tooth surrounding gingival epithelium, and it must be taken into account as one of the most important causes of bacteremia [8]. On the other hand, the sensitivity of PCR-based molecular procedures to detect oral bacteria might be high or low, depending on whether the sampling was done before or after the extraction procedure [9, 10]. However, recent studies have reported that the combination of culture medium and molecular techniques is more effective than culture technique alone [11]. In the current study, the prevalence of bacteremia before, during, and after tooth extraction, via culture technique, detected 0%, 62%, and 20%, respectively. A significant difference was observed in the prevalence of bacteremia before and after tooth extraction. This difference was also significant between the amount of bacteremia during and after tooth extraction. The prevalence of bacteremia in these three time intervals by molecular techniques (PCR) was calculated at 10%, 78%, and 32%, which demonstrates an increase in the prevalence of bacteremia in relation to culture technique. The results of this study were consistent with that of Lochhart, who reported 89% and 94% bacteremia in the experimental and control groups, respectively, when evaluating the blood culture 1 and 3 minutes after tooth surgery. A significant difference was observed between positive blood cultures in short-term (less than 3 minutes) and long-term (more than 6 minutes) sampling after the surgery [3]. The identities of microorganisms evaluated in this study are in line with those found by other researchers [3, 6]. These microorganisms were chosen based on the prevalence of oral bacteria, the specificity of salivary microorganisms, plaque, or periapical abscess. Gram-positive cocci *S. mutans*, *salivarius*, *sanguinis*, *E. faecalis*, and anaerobic bacilli, including *Lactobacillus*, were chosen. Their sequences were evaluated for their detection in molecular techniques.

In previous studies, it was noted that bacteremia was negative before teeth manipulation and extraction, but it is not necessary to evaluate it [3, 7, 12]. The current study showed that considering the negative results of culture technique for the presence of bacterial strains, Multiplex PCR method could detect *Lactobacillus* and *E. faecalis* in 2 samples and *S. mutans and S. sanguinis* in 1 sample. Therefore, it is possible for bacteremia to occur before the extraction procedure. This might be due to periodontal infections and epithelial damage near the blood vessels. It was also clarified that the accuracy of molecular techniques was higher than the standard culture technique. Similar to the results of this study, in a pilot study by Benítez-Páez (2013), transient bacteria were detected in 1 out of 8 samples, 30 seconds after surgery, via culture technique, while the PCR method detected 5 positive samples [6]. Hence, clearly the bacterial variation is detected less in the culture technique than in PCR technique.

In evaluation of bacteremia during tooth extraction, culture technique could only detect *Lactobacillus* in 9 samples, *E. faecalis* in 4 samples, *S. mutans* in 4 samples, *S. sanguinis* in 2 samples, and *S. salivarius* in 1 sample, while a molecular technique could detect more strains. PCR technique detected *Lactobacillus* in 9 samples, *E. faecalis* in 9 samples, *S. mutans* in 16 samples, *S. sanguinis* in 9 samples, and *S. salivarius* in 11 samples, during tooth extraction. These data also show the higher accuracy of molecular technique in comparison with culture technique. In line with our study, a study by Bahrani-Mougeot et al. reported that molecular technique was more accurate than biochemical and culture technique in detecting bacteremia. They found 31 positive samples via biochemical technique and 10 samples by culture technique, while the positive sample detected with molecular techniques was 40 out of 58 [13]. The evaluation of samples 15 minutes after tooth extraction by culture technique showed 2 positive samples for *Lactobacillus* and *E. faecalis* and 1 positive sample for *S. sanguinis*; no *S. salivarius* was detected at this time. Molecular technique detected the same number of *Lactobacillus* and *E. faecalis*, but *S. mutans*, *S. sanguinis*, and *S. salivarius* were proliferated in 3, 2, and 3 samples, respectively. The results of the current study showed that tooth extraction causes bacteremia before, during, and after tooth extraction. Since blood circulation from the tooth socket occurs in less than 30 seconds, and that bacteremia starts with the very first manipulation of the teeth in the gingival sulcus, the intervals of 30 seconds and 15 minutes after tooth extraction were chosen for microbial analysis.

As stated in the results, in association with lactobacilli, the highest antibiotic resistance was to tetracycline and the least was to ampicillin and tigecycline. This is different from the findings of Dušková (2013) who reported the highest resistance to gentamicin and the lowest to ampicillin. The reason for this difference can be attributed to the difference in the method of study. In this study, the bacterial culture method was used, and the sample was not blood but a variety of foods [14]. In the present study, *Mutans* showed the highest resistance to tetracycline and colistin and the least to tiag and imipenem. In the El Sherbiny study (2014), the most antibiotic resistance was to ciprofloxacin, bacitracin, and methicillin and the least was to vancomycin, penicillin, and erythromycin. Also, in this study, the samples were prepared from saliva and dental plaque and a culture study, and only 10 antibiotics were examined [15]. In relation to *E. faecalis*, the highest resistance was to tetracycline, ampicillin-sulbactam, and ciprofloxacin, and the lowest resistance to piperacillin-tazobactam. In the study by Komiyama (2016) and Mitsou (2015), most resistant was to tetracycline, which was similar to our study [16, 17].

As far as we know, there is no similar study in relation to the two species of Salivaria and Sanheu. The disk diffusion technique was used to evaluate the results of antibiotic resistance in different bacterial strains. In this study, a total of 20 common antibiotics that affect the oral microflora were evaluated. The evaluation was based on the results of PCR technique and was performed in two stages during and after tooth extraction. Generally, the highest antibiotic resistance occurred against tetracycline, ciprofloxacin, and ampicillin-sulbactam. In most cases, the bacteria showed partial resistance to these antibiotics. Tigecycline exhibited 100% efficacy on all types of bacteria. *Streptococcus* strains (*salivarius*, *mutans*, and *sanguinis*) were sensitive to most of the antibiotics while antibiotic sensitivity was less evident in *Lactobacillus* and *E. faecalis*.

## 5. Conclusion

The results of the current study showed that tooth extraction causes bacteremia before, during, and after tooth extraction. Furthermore, the data showed higher accuracy in the molecular technique, especially Multiplex PCR, in comparison with culture technique. Generally, the highest antibiotic resistance occurred against tetracycline, ciprofloxacin, and ampicillin-sulbactam. In most cases, the bacteria showed partial resistance to these antibiotics; however, tigecycline showed 100% efficacy on all types of bacteria. *Streptococcus* strains (*salivarius*, *mutans*, and *sanguinis*) were sensitive to most of the antibiotics while antibiotic sensitivity was less evident in *Lactobacillus* and *E. faecalis*.

## Figures and Tables

**Table 1:** Primer sequences of the evaluated bacteria.

*S. salivarius* (534 bp)	Forward primer	MKK-GTGTTGCCACATCTTCACTCGCTTCGG
	Reverse primer	MKK- CGTTGATGTGCTTGAAAGGGCACCATT

*S. mutans* (433 bp)	Forward primer	5-GGCACCACAACATTGGGAAGCTCAGTT
	Reverse primer	5-GGAATGGCCGCTAAGTCAACAGGAT

*S. sanguinis* (313 bp)	Forward primer	GGATAGTGGCTCAGGGCAGCCAGTT
	Reverse primer	GAACAGTTGCTGGACTTGCTTGTC

*Lactobacillus* (667 bp)	Forward primer	AGAGTTTGATTGGCTCAG
	Reverse primer	CACCGCTACACATGGAG

*E. faecalis* (941 bp)	Forward primer	5-ATC AAG TAC AGT TAG TCT TTA G-3
	Reverse primer	5-ACG ATT CAA AGC TAA CTG AAT CAG T-3

**Table 2:** Evaluated antibiotics.

1	Colistin (CO, 10 *μ*g)
2	Rifampin
3	Ampicillin sulbactam (SAM, 20 *μ*g)
4	Piperacillin (PIPRA, 100 *μ*g)
5	Piperacillin-tazobactam (PI+TZ, 100 + 10 *μ*g)
6	Ticarcillin-clavulanate (TIM, 85 *μ*g)
7	Ceftriaxone (CTR, 30 *μ*g)
8	Amikacin (AMI, 30 *μ*g)
9	Minocycline (MIN, 30 *μ*g)
11	Tetracycline (TET, 30 *μ*g)
12	Ciprofloxacin (CIPR, 5 *μ*g)
13	Ceftazidime (CAZ, 30 *μ*g)
14	Cefotaxime (CTX, 30 *μ*g)
15	Cefepime (FEP, 30 *μ*g)
16	Ertapenem (ETP, 10 *μ*g)
17	Meropenem (MRP, 10 *μ*g)
18	Imipenem (IMP, *μ*g)
19	Cotrimoxazole (SXT, 25 *μ*g)
20	Tigecycline (10 *μ*g)

## Data Availability

The experimental and clinical data used to support the findings of this study are included within the article.
